# The Early Prevention of Obesity in CHildren (EPOCH) Collaboration - an Individual Patient Data Prospective Meta-Analysis

**DOI:** 10.1186/1471-2458-10-728

**Published:** 2010-11-25

**Authors:** Lisa M Askie, Louise A Baur, Karen Campbell, Lynne A Daniels, Kylie Hesketh, Anthea Magarey, Seema Mihrshahi, Chris Rissel, John Simes, Barry Taylor, Rachael Taylor, Merryn Voysey, Li Ming Wen

**Affiliations:** 1 NHMRC Clinical Trials Centre, University of Sydney, Sydney, NSW, Australia; 2 Discipline of Paediatrics and Child Health, University of Sydney, Sydney, NSW, Australia; 3 School of Exercise and Nutrition Sciences, Deakin University, Melbourne, VIC, Australia; 4 School of Public Health, Queensland University of Technology, Brisbane, QLD, Australia; 5 Department of Nutrition and Dietetics, Flinders University, Adelaide, SA, Australia; 6 Health Promotion Service, Sydney South West Area Health Service, Sydney, NSW, Australia; 7 Department of Women's and Children's Health, University of Otago, Dunedin, New Zealand; 8 Edgar National Centre for Diabetes and Obesity Research, Medical and Surgical Sciences, University of Otago, Dunedin, New Zealand

## Abstract

**Background:**

Efforts to prevent the development of overweight and obesity have increasingly focused early in the life course as we recognise that both metabolic and behavioural patterns are often established within the first few years of life. Randomised controlled trials (RCTs) of interventions are even more powerful when, with forethought, they are synthesised into an individual patient data (IPD) prospective meta-analysis (PMA). An IPD PMA is a unique research design where several trials are identified for inclusion in an analysis before any of the individual trial results become known and the data are provided for each randomised patient. This methodology minimises the publication and selection bias often associated with a retrospective meta-analysis by allowing hypotheses, analysis methods and selection criteria to be specified *a priori*.

**Methods/Design:**

The Early Prevention of Obesity in CHildren (EPOCH) Collaboration was formed in 2009. The main objective of the EPOCH Collaboration is to determine if early intervention for childhood obesity impacts on body mass index (BMI) z scores at age 18-24 months. Additional research questions will focus on whether early intervention has an impact on children's dietary quality, TV viewing time, duration of breastfeeding and parenting styles. This protocol includes the hypotheses, inclusion criteria and outcome measures to be used in the IPD PMA. The sample size of the combined dataset at final outcome assessment (approximately 1800 infants) will allow greater precision when exploring differences in the effect of early intervention with respect to pre-specified participant- and intervention-level characteristics.

**Discussion:**

Finalisation of the data collection procedures and analysis plans will be complete by the end of 2010. Data collection and analysis will occur during 2011-2012 and results should be available by 2013.

**Trial registration number:**

ACTRN12610000789066

## Background

Primary prevention of childhood overweight is an international priority [1]. In Australia in 2007, 18-21% of 2-8 year olds were already overweight, including 4-6% who were obese [2]. Corresponding figures for New Zealand children aged 2-14 years show 21% overweight, including 8% who were obese [3]. Childhood overweight has an immediate negative impact on physical and psychological health and well-being [4,5]. In addition, overweight children have a substantially increased risk of becoming overweight adults, with an attendant increased risk of morbidity and mortality [6,7].

Childhood obesity prevention studies to date have largely targeted older children in school or community settings, and have had limited effectiveness, partly due to design and methodological issues [8-10]. The 2005 Cochrane Review of interventions for preventing childhood obesity included 22 quality trials, only three of which included children less than five years of age [10]. A more recent systematic review of the effect of interventions on weight status of children aged from birth to five years identified seven of 22 studies that included children less than two years of age [9]. However, only two of these seven studies provided growth data and hence the majority of studies in preschool aged children provide impact evaluation rather than outcome evaluation of the direct effect of interventions on weight status. Given that so few studies have focused on very young children, it is likely that existing interventions have started after feeding, eating, sleeping, television (TV) viewing and activity patterns have been established and are more difficult to modify.

There are numerous reasons why starting interventions to prevent childhood obesity very early may be effective. Rapid early weight gain before two years of age is associated with increased risk of overweight in later childhood [11-13] and most excess weight gained before puberty is gained by the age of five years (91% girls, 70% boys) [14]. A range of potentially modifiable factors operating early in life are also likely to be linked with later obesity or obesity-promoting behaviours. These are summarised below.

### Dietary patterns and eating habits

A meta-analysis has shown that children who were breastfed have significantly lower levels of obesity than those who were formula fed [15] although this association remains controversial [16]. The types and texture of foods offered to infants as they transition from milk to family foods are key determinants of early food preferences, intake patterns and dietary quality [17-19] which then track to older ages and are associated with later obesity risk [20].

### Parental early feeding practices

Parental feeding practices strongly influence children's eating behaviours, which are firmly established by five years of age and lay the foundation of adult eating habits [21,22]. Parental feeding practices determine infant exposure to food (type, amount, frequency), and include responses (e.g. coercion) to infant feeding behaviour (e.g. food refusal). The degree of parental control in child feeding (including restriction, monitoring or pressure) is associated with later child feeding behaviour (preferences and intake) and weight status [23].

### Physical activity

Most studies show an inverse relationship between physical activity levels and adiposity in children. High levels of physical activity reduce the likelihood of weight gain over time [24,25]. A systematic review of strategies to reduce obesity in children has shown that changes in weight occur most frequently in interventions that also demonstrate change in physical activity levels [26]. However, the systematic review noted that only 3% of studies (n = 5) were conducted in preschool settings.

### TV viewing practices and sedentary behaviours

Children who watch TV for more than two hours per day are more likely to be obese, have unhealthy dietary patterns and low levels of physical activity [27]. Many young children exceed this viewing threshold and such patterns of sedentary behaviour track throughout childhood [2,28].

### Sleep patterns

Numerous cross-sectional and prospective studies have linked shorter sleep duration with obesity [29-31] and increased cardiovascular risk [32]. Studies are urgently required to determine the effects of altering sleep patterns on body weight during childhood.

### Parenting style

Emerging evidence has linked parenting style (e.g. authoritative, authoritarian, libertarian) to early feeding practices, child eating behaviour and weight status. A recent review concluded that the majority of the evidence is cross-sectional, with only seven of 67 studies providing longitudinal follow-up, and none including children aged less than five years [23]. Only two studies (both in preschools) examined whether parenting feeding practices can be modified and most studies failed to evaluate covariates, particularly maternal weight status and family socioeconomic status.

### Other potential effect modifiers

A range of other factors also influence the development of excess weight gain in childhood and may, in turn, modify responses to obesity prevention interventions. These include birth weight (both low *and *high birth weight) [33]; lower socioeconomic status [34]; parental, and especially maternal, body mass index (BMI) [35]; and maternal smoking during pregnancy [35].

Overall, there is a strong argument for interventions that start early in childhood to be effective in preventing childhood obesity. However, there are as yet no published trials that provide quality evidence to guide the design, content and implementation of effective interventions that target infants. There are currently four randomised controlled trials underway in Australia and New Zealand (Healthy Beginnings [36], Nourish [37], The Melbourne InFANT Program [38] and POI.NZ [39]) that are investigating the effects of early intervention in preventing childhood obesity. The trials are similar with respect to design, participants, intervention, comparators and outcome measures. Table 1 gives the details of each trial.

**Table 1:** Characteristics of studies included in EPOCH Collaboration

Trial name	**Healthy Beginnings**^**33 **^**(RCT)**	**Nourish**^**34 **^**(RCT)**	**The Melbourne InFANT Program**^**35 **^**(cluster RCT)**	**POI NZ**^**36 **^**(2×2 Factorial RCT)**
Registration number	ACTRN12607000168459	ACTRN12608000056392	ISRCTN81847050	NCT00892983

Funding source	NHMRC	NHMRC	NHMRC	HRC New Zealand

Number randomised	N = 667 first time mothers	N = 698 first time mothers	N = 559 first time mothers	N = 400 first time mothers

Baseline data	Antenatal	Infant aged 4-6 months	Infant aged 3 months	Antenatal

Primary outcome	Child weight and height at 24 months	Child weight and height at 24 months	Child weight and height at 18 months	Child weight and height at 24 months

Usual care	Wk1 child health nurse home visit Self access to child health clinics	Self directed access to child health clinics	Access to 10 scheduled child health clinic visits	First 4 wks midwife home visits; Well Child (Plunkett) nurse: 8 visits in 5 yrs

Intervention mode	Home visits; 8 visits over 2 years (antenatal, 1, 3, 5, 9, 12, 18, 24 months); maternal advice	Two education peer support modules (6 fortnightly sessions each) commencing when infant 4-7 months and again at 13-16 months at community health venues	Six 2 hour sessions delivered at 3 monthly intervals within pre-existing mothers groups, commencing at 3 months (3, 6, 9, 12, 15 and 18 months)	3 groups: sleep (home visits at 3 wks, 4 months); healthy eating and activity (mix of 7 home visits and group based sessions at 1 wk + 3, 4, 7, 9, 12, 18 months) or both

Intervention content (in addition to usual care)	Sustaining breastfeeding; Timely solid food introduction; Responsive to child cues of hunger & satiety; Healthy child food intake; Reduced TV viewing; Promote active play; The "how" of child feeding (e.g. managing food fussiness)	Neutral repeated exposure to a variety of foods; Responsive to child cues of hunger & satiety; Healthy child food intake; Reduced TV viewing; Promote authoritative parenting style; The "how" of child feeding (e.g. managing food fussiness)	Responsive to child cues of hunger & satiety; Healthy child food intake; Reduced TV viewing; Promote active play; The "how" of child feeding (e.g. managing food fussiness)	Sustaining breastfeeding; Timely solid food introduction; Responsive to child cues of hunger & satiety; Healthy child food intake; Reduced TV viewing; Promote active play; The "how" of child feeding (e.g. managing food fussiness); Developing good sleep habits

Control group	Usual care plus written home safety/tobacco prevention information at f/up sessions plus three mail outs	Usual care plus quarterly newsletter on general child health messages excluding sleep, food and activity	Usual care plus quarterly newsletter on general child health messages excluding sleep, food and activity	Usual care

The Early Prevention of Obesity in CHildren (EPOCH) collaboration was formed in 2009 with the objective of conducting an IPD PMA of these trials to provide the necessary evidence regarding the efficacy of interventions to reduce obesity-related risk factors early in infancy. The key feature of this prospective collaboration is to define and clearly specify the objectives, research questions, specific aims, hypotheses, subject eligibility criteria, subgroups, predictors, outcomes (primary and secondary) and the general analysis plans of eligible studies in advance of knowing or publishing individual trial results [40]. IPD PMA provides more reliable estimates of treatment effects through prospectively planned combined analyses of randomised controlled trials.

While the individual trials in the EPOCH Collaboration will provide important data in their own right, combining such data via an IPD PMA will yield even more powerful information. This is particularly important in this area as any one trial, even with many hundreds of children, may alone not have sufficient statistical power to detect an overall reduction in population-relevant outcomes. For example, to show a reduction in the prevalence of overweight/obesity at age two years from 20% to 15%, 1800 participants would be required. At the current recruitment and retention rates, the combined data from the four EPOCH trials will yield a final sample size of 1810 children by age two years. The additional power conferred by the PMA, combined with the availability of the IPD, will enable a range of subgroup analyses to be performed, which will explore other factors that may be associated with obesity in children and may modify intervention effects. The information obtained from the EPOCH Collaboration will be used to guide decision making regarding investment in child health interventions which are effective in reducing the prevalence of childhood obesity and associated negative short- and long-term health outcomes.

## Methods/Design

### Objectives

The main questions that will be addressed by the EPOCH Collaboration are:

1. Do early intervention programs designed to prevent childhood obesity, compared with usual care, offer clinically important benefits in terms of lower BMI z scores at age 18-24 months; higher prevalence of breastfeeding; better children's dietary quality; less child TV viewing time and higher prevalence of parenting styles and feeding practices that are consistent with effective self regulation and development of healthy weight status?

2. Do the effects of early interventions to prevent childhood obesity differ according to the risk profile of the infants and their families in terms of birth weight, maternal education, maternal BMI and maternal smoking status?

3. Do the intervention effects differ according to a) mode of delivery (home, clinic or community-based), b) intensity (number/frequency of sessions), c) whether the intervention commenced antenatally or after birth, and d) the extent of 'well-child' early childhood services already offered in the community?

### Search methods for identification of studies

Efforts to identify ongoing trials within Australia and New Zealand that were eligible for participation in this PMA included searches for published protocols on online databases such as MEDLINE, EMBASE and clinical trial registries, as well as internet searches for media articles, non-peer reviewed articles and other publications using Google. Further efforts included informing networks of the proposed PMA and approaching presenters at relevant conferences and meetings. Four randomised controlled trials (RCTs) were identified for inclusion according to the criteria specified below and the EPOCH Collaboration was formed in 2009. The EPOCH Collaboration comprises the investigators of the four RCTs assessing childhood obesity prevention interventions in Australasian settings and a coordination/methodology team. These trials are the first internationally to address this issue and the only trials in Australasia assessing obesity prevention interventions in early infancy. The four participating trials are all funded separately by the Australian National Health and Medical Research Council (NHMRC) or the New Zealand Health Research Council. Details of the four RCTs are given in Table 1.

### Eligibility criteria for included RCTs

#### Eligibility criteria for trial design

To be eligible for inclusion in this IPD PMA, each trial had to be randomised with an adequate level of allocation concealment, as outlined in the Cochrane Handbook for Systematic Reviews of Interventions [41]. A minimum planned follow-up of infants to 18 months of age was required within each trial, and the trial had to be conducted within Australia or New Zealand. All participating trials had to be be registered on a publicly accessible clinical trial registry. To be considered for inclusion in the PMA, the participating investigators had to be blinded to their trial's outcome data by intervention group at the time the PMA objectives, research questions, specific aims, hypotheses, subject eligibility criteria, subgroups, predictors, outcomes (primary and secondary) and general analysis plans were agreed upon.

#### Eligibility criteria for the patient population

The patient population for this PMA is restricted to first time mothers and their healthy term babies. Mothers and babies must be recruited into the trials prior to the child being aged six months.

#### Eligibility criteria for each intervention and comparator

The intervention in each participating trial had to be a parent-focused intervention for early childhood obesity prevention. Early interventions were to commence before the child was seven months old and be delivered, at least in part, face-to-face but could be delivered in either home, clinic or community settings.

### Power calculations, sample size and expected treatment effects

An expected final combined sample size of approximately 1800 infants will be available for this PMA. This sample size will have 80% power at the two-sided 5% level of significance to detect the following treatment effects:

1. Reduction in mean BMI z scores (SD) from 0.53 (0.93) (control group) to 0.41 (treatment group) [42]

2. Reduction in the prevalence of overweight/obese children from 20% (control) to 15% (treatment) [3]

3. Increase in the prevalence of breastfeeding at 6 months from 33% (control) to 39.5% (treatment)

4. Reduction in child TV viewing time from 58 min/day (SD 66) (control) to 49 min/day (treatment) [43]

### Main Outcomes

Infant height/length and weight will be collected, BMI calculated (weight/height^2^) and BMI z score determined in accordance with World Health Organization (WHO) growth standards [44]. The primary outcome measure will be BMI z score at age 18-24 months.

Secondary outcomes include:

• overweight/obesity prevalence

• breastfeeding duration

• child dietary quality

• child TV viewing time

• parenting styles/feeding practices

• sleeping patterns

• measures of physical activity

### Planned analyses

Continuous outcomes will be analysed using linear models and binary outcomes will be analysed using appropriate generalised linear models with adjustments for baseline values where applicable. Models will be selected which accommodate the non-independence of observations within trials and treatment clusters and adjust for these with a modelling framework (e.g. mixed effects models for continuous data and generalised estimating equations for binary data with an exchangeable correlation structure). Any time to event endpoints will be analysed using appropriate models which accommodate censored data (e.g. proportional hazards models). As a sensitivity analysis, additional analyses will be performed on the main outcomes which adjust for important baseline factors.

#### Subgroup analyses

Subgroup analyses of both participant-level and intervention-level characteristics will be performed on the primary outcome to assess if the intervention effect differs between certain groups of children/mothers. Participant-level characteristics to be assessed include birth weight, mother's educational attainment, mother's BMI and smoking during pregnancy. Intervention-level factors which may modify the treatment effect include: where the intervention is delivered (home, clinic or community); intensity/frequency of sessions; timing of intervention onset; and the presence/absence of "well-child" services already offered in the community. Any differences between treatment effects within subgroups will be assessed by examining the significance of the subgroup by intervention interaction term within the model.

Analysis will include all randomised infants with available data and be based on intention-to-treat. Missing data will be described and reasons for missing data explored. The impact of missing data on conclusions about the treatment effect on the primary outcome will be explored where possible (e.g. using sensitivity analyses or imputation techniques).

The primary analysis will be conducted at the 0.05 level of significance. Secondary analyses also conducted at the 0.05 level of significance will be used to supplement conclusions based on the primary analysis and will be interpreted appropriately within that context, considering the totality of evidence available. Subgroup analyses have been limited to a set of biologically plausible and clinically meaningful set of subgroups which will be performed on the primary endpoint only in order to limit the potential for type I error inflation. A detailed statistical analysis plan will be prepared and agreed upon by the EPOCH Collaboration members prior to final analyses.

### Project management and data collection

The EPOCH Collaboration will undertake this IPD PMA according to the methods recommended by the Cochrane Collaboration Prospective Meta-Analysis Methods Group [40].

#### Project management

Membership of the EPOCH Collaboration includes representatives from each of the trials contributing data to the project with an accompanying project coordination and data management structure including experts in the fields of IPD PMA meta-analysis, data monitoring and statistics. The project coordination team will be responsible for all communications including organising regular teleconferences, an annual face-to-face meeting and newsletter updates. The data management team will be responsible for establishing processes leading to a commonly defined, core dataset of key variables being available from each participant in each trial and the analysis of the subsequent data.

#### Data collection

The de-identified data to be collected from each of the participating trials will correspond to the minimum data required to answer the research questions. The specific format and coding required for the data will be developed and agreed upon by the EPOCH Collaborators prospectively, before the results of any trial are known. IPD for each randomised participant will then be provided by each trial. The data management team will receive and store the data in a secure, centralised, customised database at the NHMRC Clinical Trials Centre, University of Sydney. The data will be checked with respect to range, internal consistency, consistency with published reports and missing items. Trial details such as randomisation methods, and frequency and timing of the interventions will be cross-checked against any published reports, trial protocols and data collection sheets. Integrity of the randomisation process will be examined by reviewing the chronological randomisation sequence and pattern of assignment, as well as the balance of prognostic factors across treatment groups (taking into account stratification factors). Inconsistencies or missing data will be discussed with the individual trial data managers and any problems resolved by consensus. Each trial will be analysed individually and the resulting analyses and trial data will be verified by trialists before inclusion in the EPOCH Collaboration database. New variables will be created from the common core dataset, as required, in order to answer the research questions prospectively agreed by the EPOCH Collaborators.

### Ethical considerations

#### Data ownership and confidentiality

Participants in the individual trials have previously consented to participation in their respective trial. The data are available through an agreement between all Chief Investigators of the included trials and ethical approval for each of the trials has been given by their respective Human Research Ethics Committees. The trialists remain the custodians of their own data and retain the right to withdraw their data from the analysis at any time. Data will be de-identified before being shared with the EPOCH Collaboration data management team.

#### Publication policy

Each trial has the right to publish the main results of their project prior to the EPOCH Collaboration results being published. When publishing individual results, participating trials will aim to include reference to the EPOCH Collaboration within the publication. Before publication of any EPOCH manuscripts, drafts will be circulated for comment, revision and approval by a nominated representative of each of the participating trials. Publications using these data will be authored on behalf of the EPOCH Collaboration, either with specific named authors, or on behalf of the Collaboration as a whole and names of other participating Collaborators will be listed in the Acknowledgements.

#### Data monitoring procedures

Each participating trial will have its own method of monitoring data and safety. This may be in the form of a Data and Safety Monitoring Committee (DSMC) or in some cases an independent biostatistician. The Chief Investigators of each trial will seek to ensure that the Chairperson of their own DSMC/biostatistician knows of the existence of the other participating trials so that communication can occur between them if required. The EPOCH Collaboration will give consideration to any requests from DSMC Chairpersons for sharing of de-identified data (either aggregate or IPD) should the need arise. There are 'in principle' plans to update the PMA data at regular intervals if individual trials are funded for a longer term and follow-up data become available.

## Funding

Initial funding for the EPOCH Collaboration has been received from the Meat and Livestock Association of Australia and further funding will continue to be sought from relevant funding agencies. Each individual trial has received funding from their own respective funding bodies, including the National Health and Medical Research Council (Australia), Health Research Council (New Zealand) and the Sydney South West Area Health Service. Funding bodies must provide support on condition that they will not have any input into the protocol design, data collection, data analysis or in any decisions to publish the results of the EPOCH Collaboration or any of the individual trials.

## Summary

We have described the protocol for the EPOCH Collaboration which is the first individual patient data prospective meta-analysis of early childhood obesity prevention trials in the world. It will help to answer a key public health question: do interventions implemented in the first year of life prevent obesity, and influence weight status and a range of lifestyle-relevant behavioural outcomes at 18-24 months of age? We anticipate that the results will be available for publication in 2013 (see Table 2). The proposed research pushes knowledge boundaries in terms of the individual trials and the use of the innovative IPD PMA methodology. The information obtained from the EPOCH Collaboration is needed to justify investment in child health services to provide universal access to best-practice programs that are the most effective in reducing the prevalence of childhood obesity and associated negative short- and long-term health outcomes.

**Table 2:** Timeline of studies included in the EPOCH Collaboration

RCT	Country/State	Sample size	Recruitment start date	Recruitment finish date	Age at follow up	Follow up data finalised
Healthy Beginnings**^33^**	Australia (NSW)	667	June 2007	Dec 2008	2 years	June 2011

Nourish**^34^**	Australia (QLD, SA)	698	Feb 2008	April 2009	2 years	June 2011

The Melbourne InFANT Program**^35^**	Australia (VIC)	559	April 2008	March 2009	1.5 years	Nov 2010

POI NZ^36^	New Zealand	400	March 2009	Dec 2010	2 years	Dec 2012

## List of abbreviations used

EPOCH: Early Prevention of Obesity in CHildren; RCT: Randomised Controlled Trial; IPD: Individual Patient Data; PMA: Prospective Meta-Analysis; IPD PMA: Individual Patient Data Prospective Meta-Analysis; TV: Television; NHMRC: National Health and Medical Research Council (Australia); BMI: Body Mass Index; WHO: World Health Organization; DSMC: Data and Safety Monitoring Committee

## Competing interests

Several authors on this manuscript (see below) are Chief Investigators of the individual trials included in the EPOCH Collaboration. These include Healthy Beginnings [36] (LB, CR, LW), Nourish [37] (LD, AM), The Melbourne InFANT Program [38] (KC, KH) and POI.NZ [39] (BT, RT) trials. SMs postdoctoral fellowship is funded by HJ Heinz.

## Authors' contributions

This protocol document was developed by the authors in consultation with members of the EPOCH Collaboration as listed below. All members of this group were sent draft versions and invited to comment and contribute changes. All authors have read and approved the final manuscript.

Members of the EPOCH Collaboration who contributed directly to this manuscript are: LA, LB, KC, LD, KH, AM, SM, CR, JS, BT, RT, MV and LMW.

## Pre-publication history

The pre-publication history for this paper can be accessed here:

http://www.biomedcentral.com/1471-2458/10/728/prepub
